# Cochrane corner: personal protective equipment for preventing highly infectious diseases such as COVID-19 in healthcare staff

**DOI:** 10.11604/pamj.2020.37.148.24934

**Published:** 2020-10-13

**Authors:** Chinwe Juliana Iwu, Portia Jordan, Anelisa Jaca, Chidozie Declan Iwu, Lorraine Schutte, Charles Shey Wiysonge

**Affiliations:** 1 Department of Nursing and Midwifery, Faculty of Medicine and Health Sciences, Stellenbosch University, Cape Town, South Africa,; 2 Cochrane South Africa, South African Medical Research Council, Cape Town, South Africa,; 3 School of Health Systems and Public Health, Faculty of Health Sciences, University of Pretoria, Pretoria, South Africa,; 4 Department of Global Health, Faculty of Medicine and Health Sciences, Stellenbosch University, Cape Town, South Africa,; 5 School of Public Health and Family Medicine, Faculty of Health Sciences, University of Cape Town, Cape Town, South Africa

**Keywords:** COVID-19, infectious diseases, personal protective equipment, healthcare workers, training, Africa

## Abstract

As coronavirus disease (COVID-19) cases continue to increase in Africa, healthcare workers (HCWs) have a high risk of being infected and the risks may be higher among those who work closely with patients. The risks of HCW infections can be mitigated with adequate precautions within healthcare facilities, especially with the use of personal protective equipment (PPE). We highlight and contextualise the findings of a Cochrane review on the type of PPE that protects best, the best way to put PPE on (donning) or to remove PPE (doffing) and how to train HCWs to use PPE. The review found low-certainty of evidence that full body PPE offer more protection, but HCWs may be faced with difficulty during donning and doffing. Following standard guidelines may be helpful in reducing infection and increasing compliance among HCWs. Video training and simulations may be better methods for training on the correct use of PPE than traditional methods of teaching. Countries must, therefore, ensure that HCWs undergo compulsory training on the correct use of PPE; regardless of their professional category. Of the 24 studies included in this review, none was conducted on the African continent. There is thus an urgent need for well conducted studies on the experiences of HCWs using full-body covering PPE within the African context. Such studies could lead to tailored interventions that will improve the proper use of PPE among HCWs.

## Commentary

As COVID-19 cases continue to increase in Africa, healthcare workers (HCWs) are prone to be infected and the risks may be higher among those who work closely to patients [1]. Infections among HCWs can occur when splashes or droplets of contaminated body fluids are transferred to the mucous membranes through splashes, droplets or by touching with contaminated hands [2]. The main route of exposure to COVID-19 is through droplet transmission and contact transmission [2]. The risks of HCW infections can be mitigated with adequate precautions within healthcare facilities, especially with the use of personal protective equipment (PPE) [1] which includes gowns, gloves, face masks, face shield, goggles among others. Different types of PPE are recommended based on the type of activities the HCW is involved with [3]. The COVID-19 pandemic has revealed some challenges HCWs face with the use of PPE. One of these challenges include inadequate supply of PPE [4,5], prompting the extended use and recycling of PPE in some instances [4]. Lack of training on how to use PPE by HCWs has also been reported [5] especially with donning and doffing of PPE. These issues raise safety concerns as the reusing of full-body PPE like gowns, with improper donning and doffing techniques may predispose HCWs to infections. Infected HCWs can further spread these infections to their patients and the community. The Africa CDC has provided a guideline on the types of PPE to be used by different HCWs, for different clinical settings and activities [3]. Similarly, many African countries have also guidance on the use of PPE. It is therefore important to understand which type of PPE protects best, what is the best way to donn or doff PPE and the most appropriate approaches for training HCWs on how to use PPE.

In this commentary, we highlight and contextualise the findings of an updated Cochrane review by Verbeek *et al*. on the effects of PPE among HCWs [6]. The authors assessed the effects of the following among HCWs; different types of full-body PPE used by HCWs exposed to highly infectious diseases; donning and doffing methods, adherence to CDC guidance on the use of PPE; and different training procedures. The outcomes measured were contamination of skin or clothing, infection with highly infectious diseases including Ebola virus disease, severe acute respiratory syndrome and COVID-19 and compliance with standard guidance on the use of PPE. The review considered retrospective and prospective case-control, randomised and non-randomised controlled studies eligible for inclusion in the study. Studies involving all HCWs were included, except laboratory staff. The authors searched several electronic databases, including CENTRAL, MEDLINE, Embase and CINAHL up to 20^th^ of March 2020 to identify eligible studies. Two authors independently performed study selection, data extraction and risk of bias assessment [6]. A total of 24 studies comprising of 2,278 participants were included in this review, including 14 randomised controlled trials (RCT), one quasi-RCT and nine non-randomised studies. Twelve of these studies were from the United States of America, four in China and Hong Kong, two in Canada, two in the UK, one each in Australia, Germany and Russia and one was performed in three countries at the same time: France, Mexico and Peru. One study in Canada was performed during the SARS epidemic and one study in the UK was among HCW that had returned from the West-African Ebola virus disease epidemic [6]. Evidence is generally of very low certainty, because of indirectness of the evidence in simulation studies and high risk of bias (Table 1).

**Table 1:** effects of PPE for preventing highly infectious diseases in HCWs due to exposure to contaminated body fluids

Outcome	Illustrative comparative risks* (95% CI)		Grade
	Less protective (B/D)	Most protective (A)	
Number of contaminated spots (on the palm)	B=17.83	A=7.72	Very low
Number of contaminated spots (on the palm)	D=20.49	A=12.76	Very low
**Gowns versus aprons for preventing highly infectious diseases due to contact with contaminated body fluids in healthcare workers**			
**Outcome**	Illustrative comparative risks* (95% CI)		**Grade**
	**Aprons**	**Gowns**	
Contamination: individual doffing	16.98 small spots	10.28 lower	Very low
Contamination: CDC doffing	1.88 small spots	0.62 lower	Very low
**Single-step doffing compared to Centers for Disease Control and Prevention (CDC) guidance**			
**Outcome**	Anticipated absolute effects* (95% CI)		**Grade**
	**Risk with CDC standard**	**Risk with single-step doffing**	
Contamination	917 per 1000	898 per 1000 (688 to 1000)	Very low
**Doffing with double gloves compared to doffing with single gloves**			
**Outcome**	Anticipated absolute effects* (95% CI)		**Grade**
	**Risk of doffing with single gloves**	**Risk of doffing with double gloves**	
Contamination: all body parts	733 per 1000	249 per 1000 (125 to 484)	Very low
**Doffing with extra sanitation of gloves compared to standard no sanitation**			
**Outcome**	Anticipated absolute effects* (95% CI)		**Grade**
	**Risk with no sanitation**	**Risk with extra sanitation**	
Contamination: alcohol rub	667 per 1000	500 per 1000	Low
**Video-based learning compared to traditional lecture**			
**Outcome**	Anticipated absolute effects* (95% CI)		**Grade**
	**Risk with traditional lecture**	**Risk with video-based learning**	**Very low**
Skills in PPE donning	PPE donning was 47.4%	30.7% higher	Very low

Three types of PPE attire compared by number of contaminated spots: A=not permeable, not breathable; B=breathable; D=fairly permeable, not breathable

On the types of PPE, the review found that covering more of the body leads to better protection. However, they found that it is associated with increased difficulty during donning and doffing. Modified PPE, for example, those made from more breathable materials may be more comfortable. Examples of such modified PPE are gowns that have gloves attached at the cuff, such that gloves and gowns are removed together and cover the wrist area. Gowns that are modified to fit tightly at the neck may reduce contamination. Likewise, adding tabs to gloves and face masks may lead to less contamination. With regards to guidance on PPE use, the review revealed that following CDC guidance for PPE removal may reduce self-contamination. In addition, strategies like removing gown and gloves in one step, using two pairs of gloves and cleaning gloves with bleach or disinfectant (but not alcohol) may also reduce contamination. Furthermore, the authors showed that training methods like face-to-face, computer simulation and video training were beneficial, as there were fewer errors in PPE removal compared to the written materials only or traditional lecture style. All these findings were however judged by the authors to be of low certainty because of few studies reported in the review, the indirectness of the evidence due to the simulation studies and high risk of bias. This implies that these effects must be interpreted with caution and hence a need for further investigation, especially in the African settings.

## Conclusion

The Cochrane review found a low certainty of evidence that using PPE that cover most parts of the body, modified PPE and undergoing trainings could reduce infectious diseases like COVID-19, among HCWs. However, none of the studies were conducted in Africa. It is thus important that HCWs in Africa undergo compulsory training regardless of their professional category. Furthermore, it is advisable that quality PPE must be made available for HCWs, local guidelines should be clear and easy to follow and based on international guidelines and HCWS should have maximum support from their managers. It is also important to include all healthcare facility staff when such guidelines are implemented. Most importantly, there is an urgent need for well conducted studies on the experiences of HCWs using full-body covering PPE within the African context. Such studies could lead to tailored interventions that will improve the proper use of PPE among HCWs.
